# Biogenic amines determination in some traditional cheeses in West Azerbaijan province of Iran

**Published:** 2013

**Authors:** Seyed Mehdi Razavi Rohani, Javad Aliakbarlu, Ali Ehsani, Hassan Hassanzadazar

**Affiliations:** 1 *Food and Beverage Safety Research Center, Urmia University of Medical Sciences, Urmia, Iran; *; 2 *Department of Food Hygiene and Quality Control, Faculty of Veterinary, Urmia University, Urmia, Iran; *; 3 * Food and Drug Deputy, Urmia University of Medical Sciences, Urmia, Iran.*

**Keywords:** Biogenic amines, Cheese, Histamine, HPLC

## Abstract

Biogenic amines (BA) are nitrogenous compounds that possess biological activity. The source of production is the microbial decarboxylation of amino acids. This compounds are found in various types of cheese. The aim of this work was to evaluate the BA content of some traditional cheeses in West Azerbaijan province Iran. For this purpose, 70 samples of Koopeh, 10 samples of Lighvan and 5 samples of Red Salmas cheeses were obtained from local supermarkets of different cities of West Azerbaijan province. After preparation of samples, biogenic amines content was evaluated by modified HPLC method. The presence of histamine, cadaverine, putrescine and tyramine in tested cheeses were observed. Total amount of biogenic amines was highest in Red Salmas cheese with 1426.91 ppm. It followed by Lighvan cheese and Koopeh cheese with 1008.98 and 517.71 ppm, respectively. Putrescine, cadaverine, histamine and tyramine were detected in Koopeh cheese at levels up to 156.09, 282.34, 70.80, 8.48 ppm respectively. These amines were detected also in Lighvan cheese at levels up to 277.53, 342.74, 37.58, 351.12 ppm and in Red Salmas cheese samples at levels up to 438.03, 701.05, 105.21, 182.62 ppm, respectively. Large amounts of biogenic amines can indicate non hygienic conditions and contamination of used milk for cheese production.

## Introduction

Biogenic amines (BAs) are nitrogenous compounds that possess biological activity and are mainly produced by microbial decarboxylation of amino acids, particularly histidine, tyrosine, lysine, ornithine and arginine.^1^ The presence of BAs in foods is of great interest not only due to their possible toxicity, but also can be used as indicators for quality of freshness or spoilage of foods.^2^^,^^3^ These compounds include histamine, tyramine, cadaverine, putrescine, tryptamine and 2-phenyl-ethylamine, that have been found in various types of cheeses.^2^ Cheese provides an ideal environment for the production of BAs but amine concentration differs widely and depends on several factors, such as, cheese variety, storage temperature, ripening time and microbial population.^4^ Meanwhile, other factors such as, level of proteolysis (availability of free amino acids), pH, water activity, salt-in-moisture level, bacterial counts and synergistic effect between micro-organisms have large effect on production and accumulation of BAs.^5^^,^^6^ The amine-producing ability of various bacteria differ widely. The presence of micro-organisms that have amino acid decarboxylase activity, such as *lactobacilli, enterococci, micrococci* and many strains of *enterobacteriaceae*, are necessary for production of BAs.^7^ The consumption of foods with high levels of BAs may cause several problems such as nausea, respiratory disorders, hot flushes, sweating, heart palpitation, headache, bright red rash, oral burning, hypo- or hypertension, whose intensity are depended on quantitative and qualitative differences.^5^ Some BAs have been proposed as indicators of the quality of foodstuffs.^8^^,^^9^ Therefore, the presence of biogenic amines in foods is a concern in relation to both food safety and food spoilage.^1^ Many studies have been carried out for determining the biogenic amines content in different types of cheese but studies on Iranian cheese are scanty. Recently, Aliakbarlu* el al.*, determined biogenic amine contents in Iranian white brine cheese.^10^ The purpose of this work was to evaluate the BA content of some Iranian traditional cheeses in West Azerbaijan province, Iran.

## Materials and Methods

**Sampling. **Seventy samples of Koopeh, 10 samples of Lighvan and 5 samples of Red Salmas cheeses were obtained from local supermarkets of different cities of West Azerbaijan province (Urmia, Maku, Khoy, Salmas, Mahabad, Sardasht, Oshnavieh, Naghadeh, Piranshahr, Tekab, Miandoab and Shahindezh) within the same month (Table 1). The cheese samples were chosen according to commercial availability and frequency of consumption in northwest of Iran.

**Table 1:** Sample number collected from different cities of West Azerbaijan, Iran.

**Collection city**	**Type of cheese**		
	**Koopeh**	**Lighvan**	**Red Salmas**
Urmia	9	5	-
Khoy	5	5	-
Salmas	8	-	5
Maku	5	-	-
Mahabad	8	-	-
Sardasht	5	-	-
Piranshahr	5	-	-
Oshnavieh	5	-	-
Naghadeh	5	-	-
Miandoab	5	-	-
Tekab	5	-	-
Shahindezh	5	-	-

**Chemicals and equipments. **Tyramine hydrochloride, putrescine dihydrochloride and cadaverine dihydrochloride were purchased from Sigma (Sigma Aldrich Chemical Co., St. Louis, MO, USA). Acetonitrile, water, acetone and HCl, HPLC grade, 1,7-3 diaminoheptane (1,7-Dh), histamine dihydro-chloride and dansyl chloride were obtained from Merck (Merck KGaA, Darmstadt, Germany). The chromatographic system consisted of Wellchrom HPLC pump, K-1001 (Knauer, Berlin, Germany), dynamic mixing chamber, degasser (Knauer, Berlin, Germany), Wellchrom solvent organiser K-1500 (Knauer, Berlin, Germany), UV-detector K-2501 (Knauer, Berlin, Germany), autosampler ‘Triathlon’ type 900 and a computer running the software Eurochrom 2000 (Knauer, Berlin, Germany). The column was EC 150 ⁄ 4.6 Nucleodur C18 Gravity 5 µm (Silica for powerful LC separation). 

**Chromatographic conditions. **The mobile phase was consisted of acetonitrile and water and its flow-rate was 0.8 mL min^-^^1^. The peaks were detected at 254 nm.

**Sample preparation**. A method reported by Moret and Conte modified in our laboratory was used.^10^ For acid extraction, 10 g of each sample was mixed with 20 mL of 0.1 M HCl containing the internal standard (1,7-diamino-heptane, 10 mg L^-1^) and homogenized. The suspension was centrifuged at 12000 *g* for 20 min at 4 ˚C and supernatant was collected. For organic solvent extraction, 5 mL of acid extract vortexes with three portions of 5 mL butanol. The organic extracts were saturated with NaCl and pH was adjusted to 11.5 with NaOH. For derivatization, 1 mL of organic extract was mixed with 2 drops of 1 M HCl and dried under vacuum. Then, 1 mL of 0.1 M HCl, 500 μL of saturated solution of NaHCO_3_ and 1 mL of dansyl chloride solution (5 mg mL^-1^) were added. The reaction vessel was transferred to an incubator and kept at 40 ˚C under agitation for 1 hr; then, the solution was dried under vacuum and 2 mL acetonitrile was added. The solution was filtered (Varian Bond Elut C18; Varian, Inc., Palo Alto, CA, USA) and injected onto the chromatographic column. 

**Statistical analysis.** All the obtained results were analyzed by Minitab version 15 software (Minitab Inc., PA, USA) with Two-way ANOVA. A *p* value less 0.05 was considered to be statistically significant. 

## Results

Maximum and minimum values of obtained results were shown stating presence of the BAs at the high level in cheese samples. The level of BAs (histamine, cadaverine, putrescine and tyramine) tested in traditional cheeses are shown in Table 1. Total amount of BA was the highest in Red Salmas cheese with 1426.91 ppm, followed by Lighvan and Koopeh cheese with 1008.98 and 517.71 ppm, respectively. Putre-scine, cadaverine, histamine and tyramine were detected in Koopeh cheese at levels of 156.09, 282.34, 70.8 and 8.48 ppm, respectively (Table 2). The amines were detected also in Lighvan cheese at levels up to 277.53, 342.74, 37.59 and 351.12 ppm and Red Salmas cheese samples at levels up to 438.03, 701.05, 105.21 and 182.62 ppm, respectively.

**Table 2:** Levels of biogenic amines (ppm) in Koopeh, Lighvan and Red Salmas cheeses in West Azerbaijan province, Iran.

**Cheese type**	**No.**	**Putrescine**	**Cadaverine**	**Histamine**	**Tyramine**	**Total biogenic amines**
**Koopeh**						
**Average** **Max** **Min**	70	156.09 2981.64 2.34	282.34 4697.79 2.34	70.80 1102.24 2.34	8.48 2596.91 1.99	517.71
**Lighvan**						
**Average** **Max** **Min**	10	277.53 758.24 40.90	342.74 1280.87 20.86	37.59 73.05 4.51	351.12 656.51 137.18	1008.98
**Red Salmas**						
**Average** **Max** **Min**	5	438.03 843.72 72.26	701.05 1075.84 386.50	105.21 253.98 11.59	182.62 423.56 10.06	1426.91

## Discussion

Overall, in average, cadaverine had the highest level and histamine had the lowest amount, comparing with other amines in all types of tested cheeses. The presence of BA has been extensively studied in some fermented products such as cheese, meat and wine. According to most studies, various types of cheeses contain high levels of BAs.^11^^-^^16^ and Several analytical techniques including capillary electrophoresis (CE), thin-layer chromatography (TLC) gas chromatography (GC), ion exchange chromatography, enzyme-linked immunosorbent assay (ELISA) and high performance liquid chromatography (HPLC) have been proposed for the determination of BAs in various foods.^17^^-^^20^ In this study, HPLC as a suitable technique was selected. 

Several factors causes producing and accumulation of BA in cheese and other fermented foods, such as physicochemical properties (free amino acids availability, water activity, pH, salt, moisture and temperature) and presence of microorganisms.^5^^,^^21^^-^^23^ The ripening process (involving proteolysis) cause elevated amino acid availability in cheeses, especially cheeses with long ripening periods, contributing high BA concentration.^24^ Decarboxylase positive microorganism contribute in accumulation of BA by affecting free amino acids.^25^ De-carboxylase producing microorganisms includes some strains of starter lactic acid bacteria (SLAB), non-starter lactic acid bacteria (NSLAB) and/or other spontaneous microflora.^26^ According to Martuscellia *et al.*, the amount of BAs in cheese widely varies, and is depended on the type of cheese, the ripening period, the manufacturing process and micro-organisms present.^22^ The presence of specific precursor amino acids effect the quantity and quality of BA formation, and the enzymatic activity of proteases derived from micro-organisms, or from another origin are important for the production of BA in cheese. Histamine and tyramine, are the most abundant BA found in cheeses manufactured from cows’ and ewes’ milk and usually appear after 30 days of ripening and occur in high levels in mature and in mold-ripened cheeses.^22^ Putrescine and cadaverine have also been detected in large quantities in matured cheeses.^22^ In this study, as shown in Table 1, total amount of BA was higher in Red Salmas cheese compared to other cheeses (*p *< 0.05) (Lighvan and Koopeh cheese). Lighvan cheese had a higher level of BA, comparing with Koopeh cheese (*p *< 0.05), but lower than Red Salmas (*p *< 0.05). 

In Red Salmas and Koopeh cheese putrescine and cadaverine level was higher than other BA (*p *< 0.05). In Lighvan cheese with exception of histamine, the other three BA had higher content compared to other cheese (*p *< 0.05). Spoilage micro-organisms present in milk and cheese, mainly *pseudomonads, enterobacteriaceae* and *micrococcaceae*, possess active decarboxylases^27^ and lactic acid bacteria (LAB) are less active in the decarboxylation of amino acids, but in the light of the high populations reached in cheese for long ripening periods their contribution should not be disregarded. Cheese represents an ideal environment for amine production but amine concentration varies widely and depends on such factors as cheese variety, age and microflora.^28^ Large amounts of BA can indicate non hygienic conditions and contamination of used milk for cheese making. 

In conclusion, the results emphasized the necessity of controlling hygienic conditions in raw material providing and operating manufacturing process and in the indigenous bacterial population that are responsible for high production of BA in these three types of cheeses that were produced traditionally. The use of competitive adjunct cultures is suggested.
